# Dimensions of Temperament Modulate Cue-Controlled Behavior: A Study on Pavlovian to Instrumental Transfer in Horses (*Equus Caballus*)

**DOI:** 10.1371/journal.pone.0064853

**Published:** 2013-06-14

**Authors:** Léa Lansade, Etienne Coutureau, Alain Marchand, Gersende Baranger, Mathilde Valenchon, Ludovic Calandreau

**Affiliations:** 1 Institut National de la Recherche Agronomique (INRA), UMR 85, Physiologie de la Reproduction et des Comportements, Nouzilly, France; 2 CNRS, UMR 7247, Nouzilly, France; 3 Université François Rabelais de Tours, Tours, France; 4 IFCE, Nouzilly, France; 5 Institut de Neurosciences Cognitives et Intégratives d'Aquitaine (INCIA), Université de Bordeaux, Talence, France; 6 Institut de Neurosciences Cognitives et Intégratives d'Aquitaine (INCIA), CNRS, UMR 5287, Talence, France; Université Lyon, France

## Abstract

Pavlovian to instrumental transfer (PIT) is a central factor in how cues influence animal behavior. PIT refers to the capacity of a Pavlovian cue that predicts a reward to elicit or increase a response intended to obtain the same reward. In the present study, using an equine model, we assessed whether PIT occurs in hoofed domestic animals and whether its efficacy can be modulated by temperamental dimensions. To study PIT, horses were submitted to Pavlovian conditioning whereby an auditory–visual stimulus was repeatedly followed by food delivery. Then, horses were submitted to instrumental conditioning during which they learned to touch with their noses an object signaled by the experimenter in order to obtain the same reward. During the PIT test, the Pavlovian conditioned stimulus was presented to the animal in the absence of reward. At the end of the experiment, a battery of behavioral tests was performed on all animals to assess five temperamental dimensions and investigate their relationships with instrumental performance. The results indicate that PIT can be observed in horses and that its efficacy is greatly modulated by individual temperament. Indeed, individuals with a specific pattern of temperamental dimensions (i.e., higher levels of gregariousness, fearfulness, and sensory sensitivity) exhibited the strongest PIT. The demonstration of the existence of PIT in domesticated animals (i.e., horses) is important for the optimization of its use by humans and the improvement of training methods. Moreover, because PIT may be implicated in psychological phenomena, including addictive behaviors, the observation of relationships between specific temperamental dimensions and PIT efficacy may aid in identifying predisposing temperamental attributes.

## Introduction

Adaptation to environmental change is a fundamental aspect of animal behavior. In the wild, such adaptation is facilitated by cues that guide responding toward rewarding events (e.g., food) or away from punishing ones. Vital resources are accessible to domesticated animals, but external cues can still influence the behavioral adaptation to husbandry/training practices.

In the literature, Pavlovian to instrumental transfer (PIT) is seen as a central factor in how cues influence animal behavior. PIT refers to the capacity of a Pavlovian cue that predicts a reward to elicit or increase a response intended to obtain the same reward (for a review: [1]). PIT results from the interaction between two associative learning processes. The first process is Pavlovian conditioning, during which an individual learns the relationship between a stimulus and a reward; this stimulus becomes a Conditioned Stimulus (CS). The second process is that of instrumental conditioning, during which an individual learns to perform an action in order to obtain the same reward. In the PIT test, instrumental response is assessed in the presence of the CS. If PIT occurs, CS presentation will increase the instrumental response. The ability of the CS to increase the independently acquired instrumental response characterizes PIT. PIT has been interpreted as evidence that Pavlovian CSs exert motivational influence over instrumental performance.

The explanatory schema of PIT might account for many behaviors, including drug seeking and relapse in recovering individuals with drug addiction [2], framing effects in human economic decision making [3], [4], or economic behaviors such as shopping [5]. PIT is a general process in animal cognition and behavior: it has been observed in various species such as mice, pigeons, rabbits, and rats [1]. A demonstration of PIT in domestic animals could also offer important insight into their behavior and facilitate advances in animal training. Particularly, it could help to improve training practices by triggering established behaviors when primary reinforcement is unavailable or when its delivery is impractical.

Importantly, the efficacy of PIT appears to vary between individuals: some are more sensitive than others to Pavlovian cues as behavioral triggers (e.g., in humans: [6]). Such variability could be explained by differences in dimensions of temperament. Indeed, previous studies have demonstrated that learning performance in general depends on such dimensions as fearfulness (horses [7], guppies [8]), anxiety (rodents [9]), sensitivity to stress (mice [10]), emotionality (horses [11], [12]), activity (meadow voles [13], horses [7]), sensory sensitivity (horses [7]), or shyness/boldness (dogs [14]). All of these dimensions induce differences in the level of arousal, stress, or sensitivity to stimuli known to impact learning processes (for reviews of the effects of arousal and stress, see [15], [16], [17], [18]). Thus, in the present study, using an equine model, we tested whether animal temperament influences the efficacy of PIT.

The horse is a species of choice to test the possible impact of temperament on the efficacy of PIT, since a model of temperament has been developed in this animal. Five dimensions of temperament have been characterized in horses and can be measured with a battery of behavioral tests: fearfulness [19], gregariousness [20], level of activity [7], sensory sensitivity [21], and reactivity to humans [22]. This model is one of the few that is based on behavioral tests rather than on subjective questionnaires. In addition, in contrast with those of other temperamental models, these dimensions have been shown to be stable across both situations and time; these forms of stability are fundamental to the modeling of temperamental dimensions [23], [24]. These dimensions are also independent from one another and thus measure different aspects of temperament. Finally, similar dimensions have been described in many other species, including humans (for a review: [25]).

The overall aims of this research were to determine whether PIT occurs in horses and whether its magnitude depends on temperament; those aims were achieved.

## Materials and Methods

### Ethics Statement

All animal care procedures followed were in accordance with the guidelines set by the European Communities Council Directive (86/609/EEC) and with French legislation on animal research. The procedure reported in this paper was approved by the ethics committee of Val de Loire (CEEA VdL, Comité d'Ethique pour l'Expérimentation Animale du Val de Loire). The experiment was conducted under a license from the French Ministry of Agriculture (no. 37–125), and the protocol included only behavioral observations and noninvasive contacts with the horses. A minimal number of animals per group were used for statistical testing of differences. Neither injury nor pain was observed in the horses, which lived in social groups and were taken to a paddock daily. No food restriction was used during the experimental period, and only positive reinforcement was used during the learning task. At the end of the experiment, the animals returned to their normal housing arrangements.

### Experimental design

Horses were submitted to three principal stages: Pavlovian conditioning, instrumental conditioning, and a PIT test, the latter of which assessed the impact of the Pavlovian stimulus on instrumental responding during extinction. Nineteen horses were submitted to these three stages across 5 weeks. In addition, temperament tests were performed on all animals at the end of the experiment.

### Experimental animals and facilities

The study involved 19 Anglo-Arabian horses aged 12±1 months (13 female and 6 male); all were reared under the same conditions at the experimental station of the French National Stud. They were maintained on pasture with their dam until 6±1 months of age, when they were weaned. From weaning until 11 months of age, they were housed indoors. Then, they lived on pasture until the beginning of the experiment. The males were castrated around age 10 months. All horses were accustomed to being handled and haltered.

During the 5 weeks of experiment, the horses were maintained together on pasture. Five days per week, they were housed individually in inside loose boxes (6 m×3 m) with adjacent outside areas (3 m×3 m) for 4 hrs. Horses were assigned to the same loose boxes for the 5-week duration of the experiment, and they were housed on bedding made from wood shavings. A feed bucket was placed on the front door of each box. Water and hay were available ad libitum in outside areas and pasture. No food restriction was used.

### PIT training

Throughout PIT training, each horse was tested in its own loose box. In order to reduce the stress induced by social isolation, a familiar “audience horse” was placed in the box facing the horse. The other horses were kept in their outside areas and could not see the tested horse. A horse to be tested was led by a handler from the outside area to its box or the test arena, where the test began immediately. Experimenter blinding was employed in order to rule out experimenter bias.

#### Pavlovian conditioning

**Figure 1:**
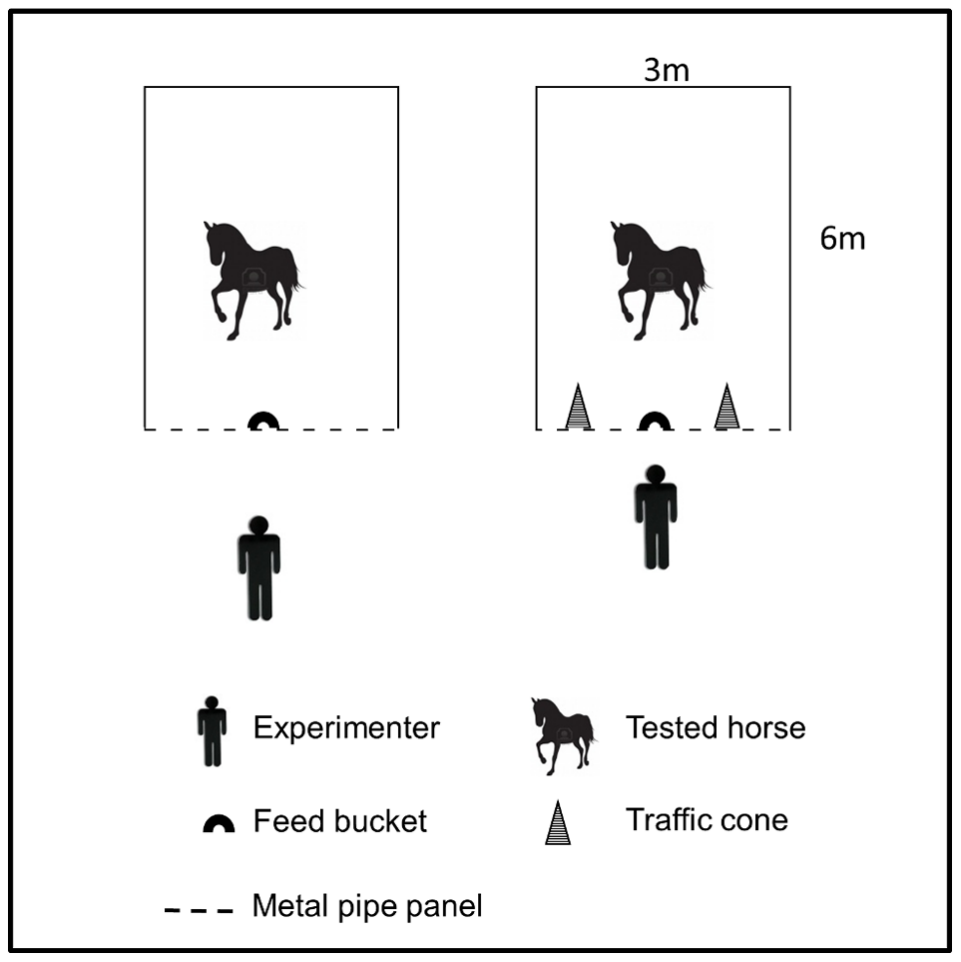
Apparatus used for Pavlovian conditioning (left), instrumental conditioning, and PIT sessions (right).

The purpose of the Pavlovian conditioning procedure was to associate an auditory–visual stimulus (the CS) with food delivery into the feed bucket. The horse could move freely in its box, and the experimenter stood 2 m in front of it (Fig. 1). The CS consisted of the experimenter shaking a 1-L plastic jug containing 500 g of pellets. The horses received a total of 9 sessions of Pavlovian conditioning training: one daily during the first 5 days and one weekly for the next 4 weeks. Each session lasted 32 min. Eight presentations of the CS were given in each session, interspersed with periods in which no stimulus was presented (intertrial interval, ITI). The length of these ITIs varied from 1.5 to 3 min (average: 2 min). The CS presentations lasted 2 min, during which the experimenter distributed small handfuls of pellets (unconditioned stimulus, UCS) into the feed bucket four times (2 s, 30 s, 1 min, and 1.5 min after the beginning of the CS). Thus, the animals received 32 distributions of pellets per session. If a horse did not eat the pellets, they were removed after 20 sec. For each session and distribution, the proportion of horses eating the pellets in less than 20 sec was recorded. The numbers of behaviors directed towards the feed bucket (licking, nibbling, or biting the feed bucket or the fence to which the bucket is attached or hitting the fence with a hoof) were also measured.

#### Instrumental conditioning

The purpose of the instrumental conditioning was to teach the horse to touch one of two cones signaled by the experimenter in order to obtain a reward (pellets). The horse could move freely in its box, and an experimenter sat 1 m in front of it (Fig. 1). The box was equipped with two orange and white traffic cones (60 cm high) fixed on both sides of the feed bucket (Fig. 1). To give the signal, the experimenter tapped the top of the cone associated with the reward with her finger for a maximum of 15 s or until the horse touched any part of it with its nose.

When a trial began, the experimenter gave the signal to the horse. If the horse did not touch a cone in 15 s, an additional 10 s were allowed to attract the horse and help it to touch the cone (with pellets or by guiding it with a halter). Immediately after the horse touched the pointed cone, a small handful of pellets were delivered into the feed bucket. Touching the other cone was not rewarded. The next trial began 10 s after the horse had swallowed the pellets and removed its nose from the feed bucket. Each session was composed of 30 consecutive trials. The experimenter pointed to the right and left cones 15 times each, ordered randomly.

The horses completed a minimum of four sessions and continued completing sessions until they reached the acquisition criterion: touching the pointed cone in 15 sec or less during 14 out of 15 consecutive trials in one session. The number of correct responses (touching the pointed cone in 15 sec or less) and the number of errors (touching the non-pointed cone) performed during each session were recorded. The number of sessions required to reach the acquisition criterion was also measured.

#### Pavlovian to instrumental transfer (PIT)

In order to optimize PIT assessment, it is necessary for the animals to exhibit a relatively low baseline level of instrumental performance. To ensure this, once a horse achieved the acquisition criterion, its instrumental performance was progressively extinguished until the horse made no more than 5 correct responses per session. To reach this criterion, the experimenter delivered the reward 75%, 50%, 25%, 17%, and 10% of the time following a correct response in the 1st, 2nd, 3rd, 4th, and 5th–8th sessions, respectively. If the performance criterion was not reached after this procedure of partial reinforcement, no reinforcement was delivered in the following sessions until the horse exhibited no more than five correct responses per session. The number of sessions required to reach the criterion was recorded.

During the PIT session, the apparatus used was the same as that described for the instrumental conditioning. The animals were tested under an extinction condition: no reinforcement was delivered during the session. The test consisted of three CS presentation periods (during which the plastic jug was constantly shaken) separated by 3 ITI periods; the test also began with an ITI. Each CS and ITI period lasted 2 min. During each of these periods, the experimenter gave five signals (tapped the pointed cone for a maximum of 15 s). Thus, there were a total of 30 trials: the left and right cones were pointed in 15 trials each, ordered randomly.

The impact of the Pavlovian stimulus on instrumental responding was determined by comparing the sum of correct responses (touching the pointed cone) during the three ITI and three CS periods. The number of correct responses was also compared between consecutive ITI and CS periods. In addition, we measured the number of errors (touching the non-pointed cone) during the ITI and CS periods.

### Temperament tests

**Figure 2:**
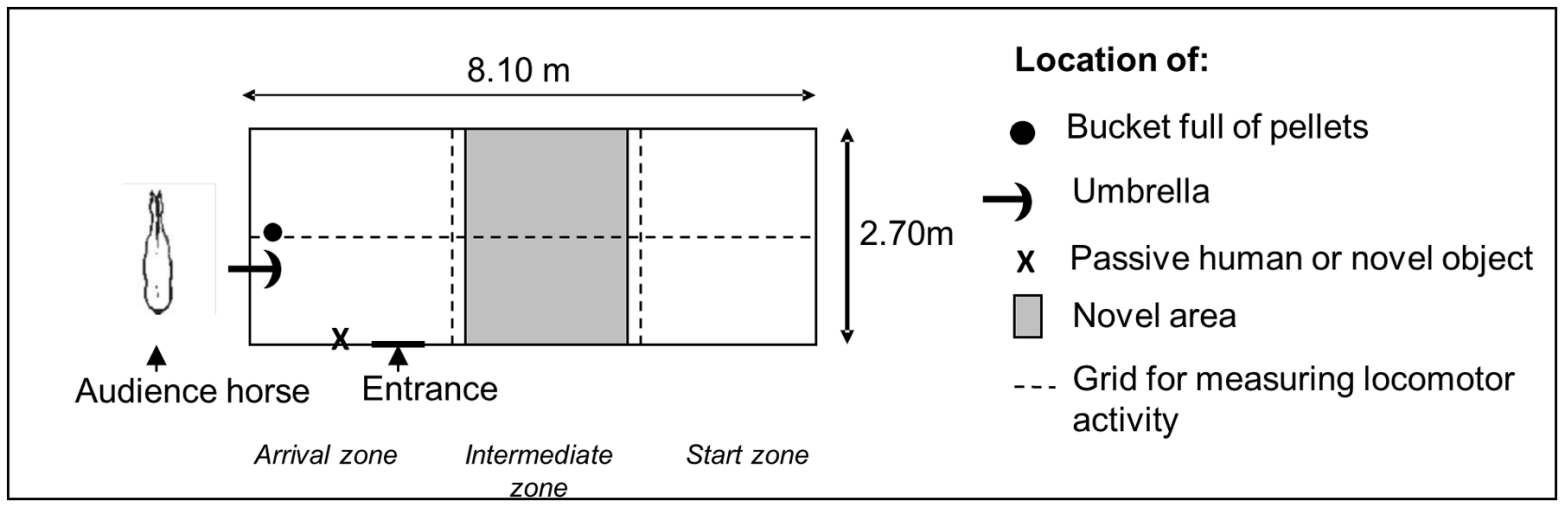
Arena used for the temperament tests.

At the end of the experiment, temperament tests developed by Lansade et al. [7], [19], [20], [21], [22] were performed. The tested horse was led into an 8.10 m×2.70 m unfamiliar test arena located in a barn adjacent to the stable (Fig. 2). Two observers were hidden behind a dark window, and an audience horse was tied up outside the box, visible to the tested horse. We analyzed the behavioral parameters selected during these studies, as they appear to be reliable indicators of horses' temperament because of their stability across time and situations. The tests are presented in the order in which they were conducted.

#### Novel arena test

The horse was led to a test arena and was left in it for 5 min. The numbers of neighs and defecations were recorded. These are considered as indicators of gregariousness and fearfulness, respectively [20].

#### Passive human test

To characterize reactivity to humans [22], an unknown experimenter (always the same person) entered the arena and remained motionless beside a wall (Fig. 2) for 3 min. The number of contacts with the human (sniffing or nibbling the experimenter) was recorded.

#### Tactile sensitivity test

An experimenter held the horse on a lunge throughout the test. A second experimenter applied a von Frey filament to the base of the horse's withers (von Frey filaments, Stoelting, IL, USA). These filaments consist of a hard plastic body connected to a nylon thread. The purpose of this test is to evaluate response to mechanical stimuli using filaments of varying strengths. Thus, the filaments are calibrated to exert specific magnitudes of force on the skin, ranging 0.008–300 g. They were applied perpendicularly to the animal's skin until the nylon filament started to bend. Trembling of the platysma muscle was recorded, and response was coded in binary form (trembling/not trembling). The test had two phases: In the first phase, which was carried out after the passive human test, a 0.008-g filament was applied to the right side of the horse, followed by the application of a 300-g filament to the left side. In the second phase, after the novel area test, a 0.02-g filament was applied to the horse's right side, followed by a 1-g filament to the left side. The frequency of responses to the filaments was recorded [21].

#### Novel object test

To characterize fearfulness [19], a novel object (colored plastic pieces attached to a 1-m–long bar) was placed near the entrance to the arena (Fig. 2) for 3 min. The number of contacts (sniffing or nibbling the experimenter) with the object was recorded.

#### Social isolation test

To characterize gregariousness [20], the audience horse was led by an experimenter outside to the barn so that it would become invisible and inaudible to the tested horse for 1.5 min. The number of neighs was recorded. However, these data were removed from analysis, because several horses showed excessively strong signs of distress (escape tendency, etc.) and did not complete the test.

#### Novel area test

For this test, which characterizes fearfulness [19], the ﬂoor of the novel arena was divided into three zones of 2.7 m×2.7 m (Fig. 2). The 1^st^–3^rd^ zones were the start, intermediate, and arrival zones (on the right, middle, and left in Fig. 2, respectively). The arrival zone contained a bucket of the pellets with which the horses were familiar. Immediately prior to the test, the horses underwent a habituation phase during which they learned to go from the start zone to the arrival zone containing the bucket. To achieve this, an experimenter led the horse by halter to the start zone and released it so that it was free to go to the arrival zone to eat. This was repeated three times. During the test, a pink carpet (2 m×2.7 m) was placed in the intermediate zone. As in the habituation phase, the experimenter released the horse in the start zone, and the time until the horse placed one hoof on the carpet was recorded. If the horse did not cross the area within 180 s, the test was terminated and a time of 181 s was assigned.

#### Suddenness test

In this test, which characterizes fearfulness [19], a black umbrella was suddenly opened in front of the animal while it was eating. A bucket of pellets was placed near the arena's entrance (Fig. 2). After the animal had been eating with its head in the bucket for 3 s, the experimenter, who was not visible to the horse, opened the umbrella. We recorded flight distance in meters.

#### Locomotor activity

In order to measure locomotor activity, we divided the test pen into six sectors of equal size (Fig. 2). We recorded the number of sectors crossed by one of the horse's front hooves during the novel arena test, the novel object test, and the passive human test.

### Statistical analysis

Data were analyzed with the XLSTAT software (Addinsoft Software, Paris, France). Because of the small number of subjects and the nature of the measurements, nonparametric statistics were used. Friedman, one-tailed Wilcoxon, and McNemar's tests were used for within-group comparisons. For acquisition, we analyzed only the sessions performed by all animals (i.e., the first four). Because many animals performed additional sessions to reach the acquisition criterion, the figure also lists the number of responses during the last acquisition session (the one during which the horses reached the criterion).

For the analyses involving temperament data, we pooled the number of responses across the first four acquisition sessions and then across the three CS presentations in the PIT session. Then, through median splits of the number of responses, we constituted groups of “low performers” and “high performers” during acquisition and “low responders” and “high responders” during PIT. We compared the temperament data between these groups with a two-tailed Mann-Whitney test.

The data are shown in box plots that illustrate median, mean, and interquartile range (IQR). The Variance of Wilcoxon (V(Ws)), McNemar's Q, Friedman Q, and Mann-Whitney U values are presented in the text. The threshold of statistical significance was set at 0.05.

## Results

### Pavlovian conditioning

The proportion of horses eating from the feed bucket at each distribution of pellets was significantly greater in the last session than the first (last session: 19/19; first session: 11/19; McNemar's test: Q = 8; *P* = 0.004). Because the horses spent the majority of their time eating during the conditioning session, very few behaviors other than eating were observed.

### Instrumental conditioning

**Figure 3:**
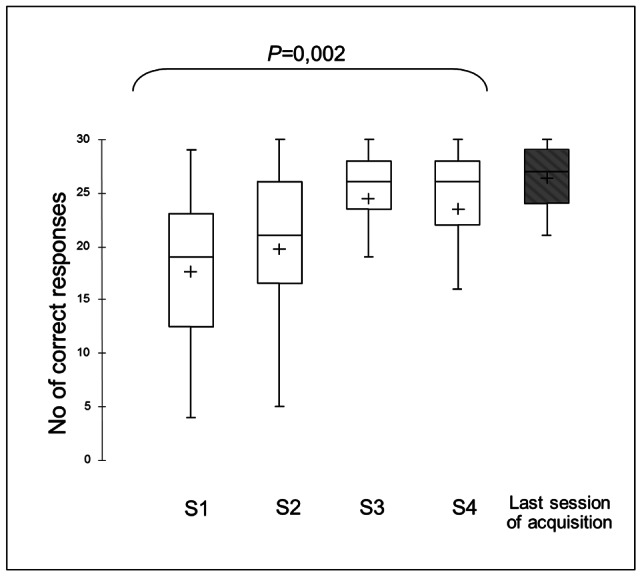
Acquisition of instrumental responding in horses. S1–S4: first 4 acquisition sessions (30 trials per session). The grey bar refers to the session during which the animals reached the criterion (touching the cone in ≤15 s during 14 out of 15 consecutive trials). Depending on the individual, this session corresponds to the 1^st^–6^th^ acquisition sessions.

The number of correct responses increased significantly across the first four acquisition sessions (Friedman test: Q = 14.95; *P* = 0.002, Fig. 3). Horses achieved the acquisition criterion between the 1^st^ and 6^th^ acquisition sessions (median [IQR]: 3[1.5;4]). Horses exhibited very few errors (touching the non-pointed cone) during acquisition (sessions 1–4, median [IQR]: 0.5[0;1], 0[0;1], 0[0;1], and 0 [0;0], respectively; Friedman test: NS).

### Pavlovian to instrumental transfer (PIT)

The criterion applied before testing PIT (no more than five correct responses per session) was achieved in the 5^th^–15^th^ sessions of partial reinforcement/extinction (median [IQR]: 8[8]; [9]).

**Figure 4:**
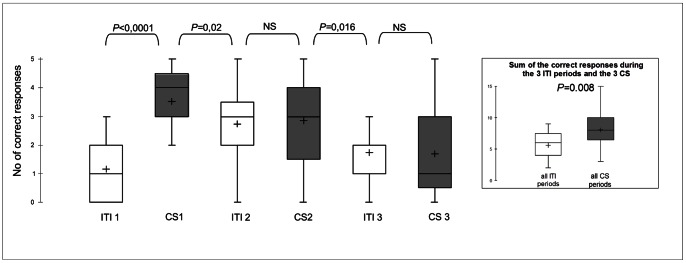
Pavlovian to instrumental transfer in horses. Left: Number of correct responses (out of 5) during the 3 presentations of the CS (CS1–CS3, in grey) as compared to the corresponding number during their respective intertrial intervals (ITI1–ITI3, in white). Right: Sum of the correct responses exhibited during the 3 ITI and 3 CS periods.

Within the PIT session, a comparison between the numbers of correct responses exhibited during the three ITI (no-CS) and three CS periods showed a significant increase in response frequency when the CS was present (Wilcoxon test: V(Ws)  = 609; *P* = 0.008, Fig. 4). There were very few errors (touching the non-pointed cone) during the ITI (median [IQR]: 0[0;1]) or CS (median [IQR]: 0[0;1]) periods (Wilcoxon test: NS).

The comparisons between consecutive ITI and CS periods showed that the animals performed more correct responses during the CS1 period than during the previous and following ITIs (Wilcoxon test: V(Ws)  = 609; *P*<0.0001, V(Ws)  = 588; *P* = 0.02, Fig. 4); more correct responses were also observed during the CS2 period than during the following ITI (Wilcoxon test: V(Ws)  = 605; *P* = 0.016, Fig. 4). The numbers of correct responses decreased between CS1 and CS2 (Wilcoxon test: V(Ws)  = 196.5; *P* = 0.04) and between CS2 and CS3 (Wilcoxon test: V(Ws)  = 193.6; *P* = 0.001).

### Influence of temperament on instrumental learning performance and PIT magnitude

**Figure 5:**
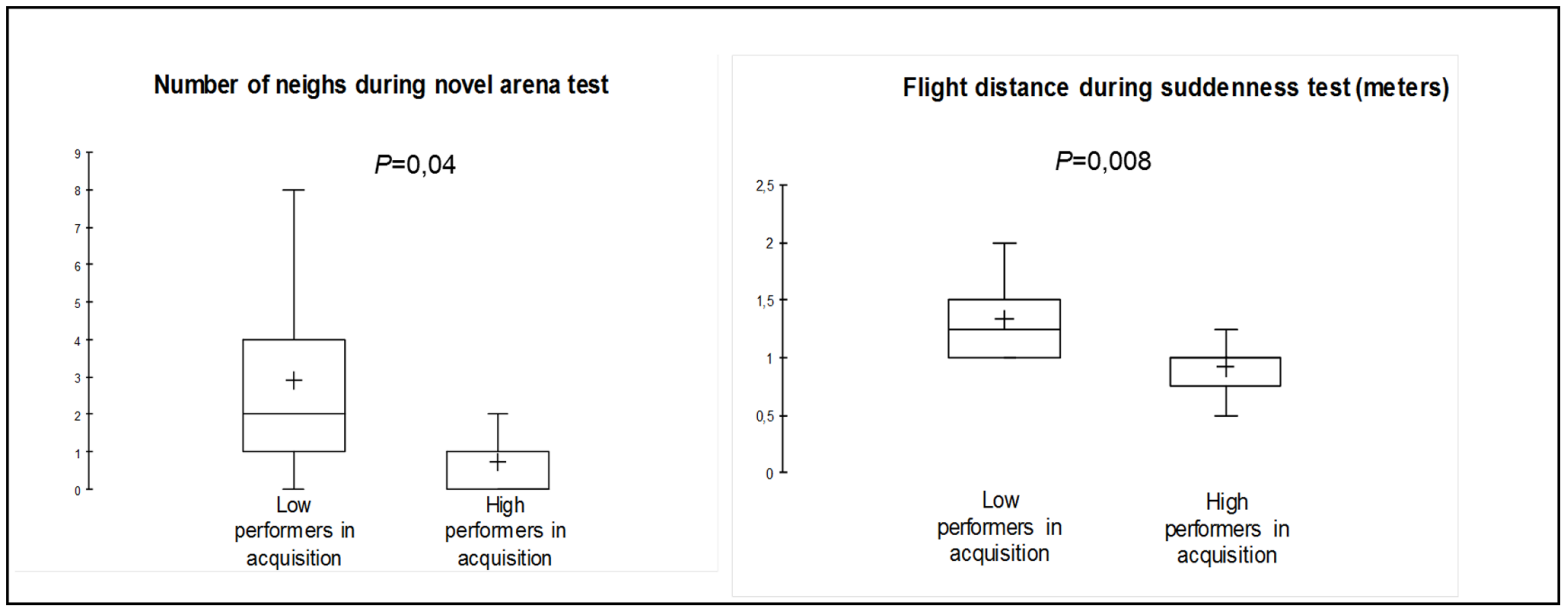
Differences in temperament between the “low performers” and “high performers” during acquisition. “Low performers”: individuals who performed the fewest correct responses during the first four acquisition sessions (N = 9). “High performers”: individuals who performed the most correct responses during the first four acquisition sessions (N = 10).

**Figure 6:**
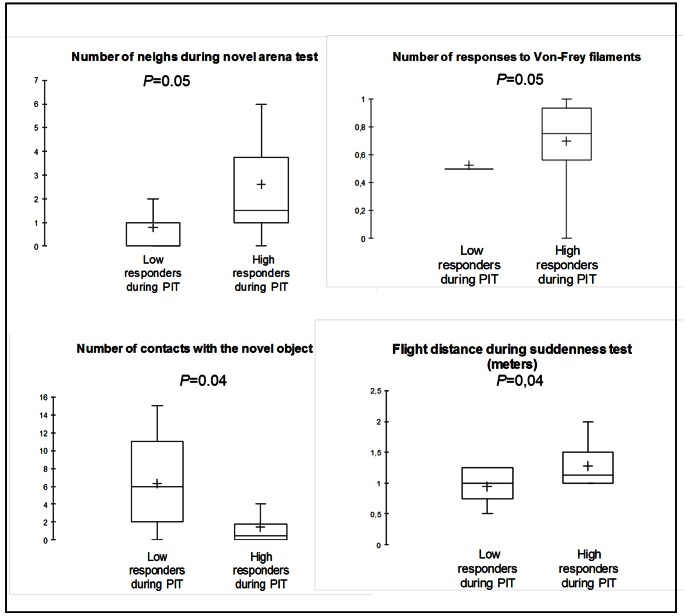
Differences in temperament between the “low responders” and “high responders” during Pavlovian to instrumental transfer. “Low responders”: individuals who performed the fewest correct responses during CS presentation (N = 9). “High responders”: individuals who performed the most correct responses during CS presentation (N = 10).

The high performers in acquisition (animals that performed the most correct responses during the first four acquisition sessions, N = 10) neighed significantly less during the novel arena test (Mann-Whitney test: U = 21.5; *P* = 0.04) and had lower flight distances during the suddenness test (Mann-Whitney test: U = 14; *P* = 0.008) than the low performers had (N = 9, Fig. 5). The high responders during the PIT session (animals that performed the most correct responses during CS presentation, N = 10) neighed significantly more during the novel arena test (Mann-Whitney test: U = 22.5; *P* = 0.05), responded more frequently to the von Frey filaments (Mann-Whitney test: U = 23; *P* = 0.05), were less frequently in contact with the novel object (Mann-Whitney test: U = 20.5; *P* = 0.04), and had greater flight distances during the suddenness test (Mann-Whitney test: U = 21.5; *P* = 0.04) than the low responders (N = 9, Fig. 6).

## Discussion

In the present study, using a Pavlovian to instrumental transfer (PIT) design, we demonstrated that external cues can trigger specific behaviors in horses. In addition, we found that the magnitude of PIT was significantly modulated by individual temperament. These findings have important implications, which are addressed in detail below.

The first aim of this study was to demonstrate the existence of PIT in horses. A detailed analysis of the instrumental responses measured over the three CS presentations during the PIT session indicates that the first CS was associated with the strongest transfer effect and quadrupled the number of correct responses compared with baseline. The results also show that the number of correct responses temporally decreased throughout the three CS presentations, suggesting extinction of the capacity of the CS to elicit the instrumental response. Nevertheless, throughout the entire PIT session, the horses responded significantly more frequently during the CS than the ITI periods. Therefore, to our knowledge, this study is the first to show that a Pavlovian CS can restore an extinguished instrumental response in horses and thus demonstrate PIT in this species.

The present results also revealed that instrumental responses can be modulated by specific temperamental dimensions. During the acquisition phase of instrumental conditioning, the high performers were those that neighed the least in the novel arena and had lower-magnitude flight reactions in the suddenness test; these were indicative of low levels of gregariousness and fearfulness, respectively [19], [20]. Negative influences of fearfulness, anxiety, shyness/boldness, or emotional reactivity on acquisition performance have been reported in the literature frequently across many species (e.g., rats: [9], dogs: [14], horses: [7], [12], [26]). The present observation of negative relationships between acquisition performance on the one hand and the dimensions of fearfulness and gregariousness on the other may be explained in terms of individual differences in stress levels between horses, which in turn may depend on temperament. Indeed, animals high in gregariousness and fearfulness are more prone to becoming stressed during the instrumental learning procedure, which represents a novel situation and involves separation from familiar conspecifics. The fact that stress is often reported to be detrimental to learning and memory (for reviews, see [15], [16], [17], [18]) may explain the negative relationships between these temperamental dimensions and instrumental acquisition performance.

High responders during the PIT test session were those that neighed the most during the novel arena test, had greater flight distances during the suddenness test, touched the novel object less often, and reacted the most to von Frey filaments. Thus, they were more gregarious, more fearful, and more sensitive to touch, according to Lansade et al. [19], [20], [21]. This relationship between a specific pattern of temperamental dimensions and PIT efficacy has never been shown before. Because PIT involves an interaction between Pavlovian and instrumental processes [1], the effects of these specific temperamental dimensions on PIT efficacy might be explained by their possible influences on each of these processes.

First, the influence of these behavioral dimensions on the magnitude of PIT may be due to their effects on Pavlovian processes. Dimensions such as gregariousness and fearfulness might have enhanced the motivational value of the CS during Pavlovian conditioning. Animals that are more prone to becoming stressed under social isolation or when facing novelty might have established a stronger CS-reward association than other horses. In line with this view, previous studies reported a strengthening impact of stress on Pavlovian conditioning [17]. Consequently, the capacity of the CS to trigger an instrumental response would result in a higher PIT magnitude in these horses. Particularly, previous studies indicated dendritic hypertrophy in the basolateral complex of the amygdala or increased reactivity of amygdala neurons that potentiate emotionality or emotional learning in stressed animals [27], [28]. The present results indicate that high trait fearfulness and gregariousness enhance Pavlovian conditioning but disrupt instrumental learning. These findings (and previous studies that indicated modulation of stress by memory systems [17]) suggest that given temperamental profiles might favor or disrupt learning and memory, depending on the cognitive processes engaged in the task.

The relationship between reaction to von Frey filaments and PIT magnitude can be explained along the same line. Indeed, the response to von Frey filaments reflects a dimension of temperament called sensory sensitivity [21]. According to Dunn [29], high–sensory-sensitivity humans notice sensory stimuli quite readily and perceive more sensory events than others; such people are easily distracted by movements, sounds, or smells. Horses with high sensory sensitivity may have noticed the CS more easily, facilitating the incentivization of this CS. Accordingly, Talmi et al. [6] described that PIT was larger when participants were aware of the presence of the Pavlovian CS.

Several studies have reported individual variations among rats in the propensity to attribute incentive motivational properties to reward cues. Such variability may partially explain the present observation of a specific pattern of behavioral dimensions on PIT performance. These studies showed that when a cue was paired with a food or cocaine reward, rats bred for high reactivity to novel environments learned to approach the cue, whereas rats bred for low reactivity to novelty learned to approach the location of food delivery. These results indicated that only rats selected for high reactivity to novelty attributed incentive value to the rewarded cue [30]. Animals that attribute greater incentive value to rewarded cues also work harder to obtain rewards [31] and are more prone to drug-cue–controlled behaviors [32]. Together, these findings suggest that individual variation in the propensity to attribute incentive value to reward cues or form Pavlovian CS-UCS associations may explain the influence of specific behavioral dimensions, such as novelty seeking or sensitivity seeking, in the modulation of PIT efficacy.

The second explanation for the influence of this pattern of temperament on PIT efficacy may be that these dimensions of temperament affect instrumental processes. Recent research in humans and rats suggests that stress favors habitual process during instrumental conditioning. This was demonstrated by Schwabe & Wolf [33] and Dias-Ferreira et al. [34], who have subjected individuals to acute or chronic stress before instrumental learning. When tested in a devaluation experiment, stressed individuals were less sensitive to change in the value of the outcome compared with non-stressed individuals, suggesting habitual rather than goal-directed behavior (for a review: [35]). Thus, in the present study, because of the higher level of stress imposed during instrumental training, the most fearful and gregarious animals may have preferentially formed habitual responses. Furthermore, it seems that PIT is stronger when the behavior is controlled by habitual (rather than goal-directed) processes. This was demonstrated by Holland [36], who found that extended training of an instrumental response known to favor habitual processes enhances susceptibility to the facilitatory effects of Pavlovian CS during PIT (for a review: [37]). Following this reasoning, we suspect that the most fearful and gregarious individuals would be more sensitive to the facilitating effect of CS during PIT, because they would preferentially form habitual responses.

Finally, we postulate that individuals with a fearful, gregarious, and sensitive temperament profile exhibited stronger PIT response because this profile confers specific constitutive cognitive abilities to horses independently of the emotion felt during the learning processes. Indeed, previous studies in rodents showed an impact of anxiety trait on cognitive performance [38], [39]. As hypothesized for these lines of rodents, good PIT performance in fearful, gregarious, and sensitive horses could be mainly cognitively driven. Indeed, this temperament profile could be associated with enhanced or more-accurate processing of environmental cues. This cognitive ability would result in both enhanced PIT performance when a CS is presented during the transfer test and strengthened emotional responses during temperament tests. Continuing research may provide a better understanding of the relationship between temperamental traits and cognitive performances.

The demonstration of the existence of a Pavlovian to Instrumental Transfer in a domesticated animal (i.e., horses) could optimize its use by humans through improvement in training methods. Especially, PIT could be useful to elicit previously established behavior when the delivery of reinforcement is impractical. It could also provide a better understanding of existing training methods in which PIT is probably involved, such as clicker training [40]. Moreover, this experiment shows that the influence of external cues on individual behavior depends on temperament. The study of the influence of temperament on sensitivity to external cues could be further developed in several species and in different contexts involving PIT, such as addiction, shopping behavior, and animal training, in order to identify predisposing factors to the presently described effects.
